# Women with adenomyosis are at higher risks of endometrial and thyroid cancers: A population-based historical cohort study

**DOI:** 10.1371/journal.pone.0194011

**Published:** 2018-03-09

**Authors:** Chih-Ching Yeh, Fu-Hsiung Su, Chii-Ruey Tzeng, Chih-Hsin Muo, Wen-Chang Wang

**Affiliations:** 1 School of Public Health, College of Public Health, Taipei Medical University, Taipei, Taiwan; 2 Department of Public Health, China Medical University, Taichung, Taiwan; 3 School of Medicine, College of Medicine, Fu Jen Catholic University, New Taipei City, Taiwan; 4 Division of Family Medicine, Department of Community Medicine and Long Term Care, Fu Jen Catholic University Hospital, New Taipei City, Taiwan; 5 Center for Reproductive Medicine and Sciences, Department of Obstetrics and Gynecology, Taipei Medical University Hospital, Taipei, Taiwan; 6 Department of Obstetrics and Gynecology, School of Medicine, College of Medicine, Taipei Medical University, Taipei, Taiwan; 7 College of Medicine, China Medical University, Taichung, Taiwan; 8 Management Office for Health Data, China Medical University Hospital, Taichung, Taiwan; 9 The Ph.D. Program for Translational Medicine, College of Medical Science and Technology, Taipei Medical University, Taipei, Taiwan; Michigan State University, UNITED STATES

## Abstract

**Objective:**

Both adenomyosis and endometriosis are characterized by the presence of ectopic endometrial glands and stroma and have been suggested to share some characteristics with malignant tumors. Although accumulating evidence indicates that endometriosis is associated with some cancer types, the cancer risks in patients with adenomyosis have been rarely examined. In this study, we investigated the relationship between adenomyosis and risks of common cancers.

**Methods:**

This study included a cohort of 12,447 women with adenomyosis but not endometriosis, born in 1951–1984, and a cohort of 124,470 adenomyosis-free women matched by birth year. Their medical records (collected between 1996 and 2011) were obtained from the National Health Insurance Research Database of Taiwan. We first compared the distribution of cancer-free survival (CFS) between cohorts with and without adenomyosis. Subsequently, within the adenomyosis cohort, we examined whether time-to-onset of the identified cancer type was correlated with time-to-onset of adenomyosis. The Cox proportional hazards model was used to compare the distribution of CFS between the adenomyosis and adenomyosis-free cohorts and between the early- and late-diagnosed adenomyosis groups. For comparison, we further evaluated the cancer risks for a cohort of 10,962 women with endometriosis but not adenomyosis and a birth-year matched cohort of 109,620 endometriosis-free women.

**Results:**

Compared with adenomyosis-free women, patients with adenomyosis had higher risks of endometrial and thyroid cancers, with estimated hazard ratios (HRs) (95% confidence interval) of 2.19 (1.51–3.16) and 1.70 (1.29–2.24), respectively. For both cancers, distributions of CFS were not significantly different between the early- and late-diagnosed adenomyosis groups. Furthermore, compared with endometriosis-free women, patients with endometriosis had higher risks of endometrial and ovarian cancers, with HRs of 1.89 (1.07–3.35) and 2.01 (1.27–3.16), respectively.

**Conclusions:**

Women with adenomyosis are at higher risks of endometrial and thyroid cancers, while women with endometriosis are at higher risks of endometrial and ovarian cancers.

## Introduction

Adenomyosis and endometriosis are two common gynecologic diseases, and both are characterized by the presence of ectopic endometrial glands and stroma [1, 2]. These diseases are commonly diagnosed in reproductive-age women, and they can result in pelvic pain, dysmenorrhea, and infertility [3]. Although adenomyosis and endometriosis are generally considered benign conditions, they have been suggested to share some characteristics with malignant tumors, such as angiogenesis, abnormal tissue growth, and invasion [4, 5]. In recent years, an increasing number of studies have investigated the association of endometriosis with several cancer types [5], particularly ovarian cancer [6]. However, few studies have examined the association of adenomyosis with cancer risk [7], except for risk of endometrial cancer [8–12].

In this study, based on a large nationwide database collected in Taiwan, we investigated the relationship between adenomyosis and the risks of different cancer types by using a two-stage approach. First, we examined whether the risks of some cancer types in patients with adenomyosis are different from those in the general population. Then, for each cancer type associated with adenomyosis, we examined whether the time of cancer onset is correlated with the time of adenomyosis onset. To investigate whether cancer types associated with adenomyosis is identical to those associated with endometriosis, we also examined the association between endometriosis and different cancer types in our first-stage analysis.

## Materials and methods

This study was approved by the Institutional Review Board of China Medical University and the Research Ethics Committee of China Medical University Hospital (IRB permit number: CMU-REC-101-012).

### Data source and study cohorts

We used data from the Longitudinal Health Insurance Database 2000 (LHID2000), which is a subset of the National Health Insurance Research Database (NHIRD) in Taiwan. The NHIRD consists of the data of insurants enrolled in the National Health Insurance program, which was launched in 1995 and covers more than 99% of Taiwan’s population [13, 14]. The LHID2000 consists of the medical records (collected between 1996 and 2011) of one million beneficiaries randomly sampled from the NHIRD (http://nhird.nhri.org.tw/en/Data_Subsets.html#S3).

A total of 486,077 women were contained in the LHID2000. As shown in S1 Table, we stratified these women by their birth year, and calculated the proportions of women having at least one diagnostic record of adenomyosis (International Classification of Diseases, Ninth Revision, Clinical Modification [ICD-9-CM] code 617.0)[3] or endometriosis (ICD-9-CM codes 617.1–617.9)[3] in individual strata. According to S1 Table, we selected women born between 1951 and 1984 as the study population because more than 5% of women had a diagnosis of adenomyosis or endometriosis in each of these strata. The study population was divided into four cohorts: (1) adenomyosis cohort, (2) endometriosis cohort, (3) both adenomyosis and endometriosis cohort, and (4) adenomyosis-and-endometriosis-free cohort.

### The distributions of cancer types

The distribution of cancer types in the adenomyosis cohort was summarized in Table 1. In this study, we only analyzed cancer types for which the patient numbers were more than or equal to 10. For each cancer type, the patients were further classified into four subsets, according to the order of diagnoses of cancer and adenomyosis (adenomyosis diagnosed before cancer or cancer diagnosed before adenomyosis) and the interval between these two events (within or more than 6 months). For a disease, the time of diagnosis was defined as the time of its first diagnostic record in the LHID2000. Similarly, the distribution of cancer types in the endometriosis cohort was summarized in S2 Table.

**Table 1:** Distribution of cancer types in the adenomyosis cohort.

Site of cancer (ICD-9-CM)	Number of	Adenomyosis diagnosed before cancer				Cancer diagnosed before adenomyosis			
	cancer	More than 6 months		Within 6 months		Within 6 months		More than 6 months	
	patients	n	%	n	%	n	%	n	%
Head and neck (140–149)	24	13	54	0	0	0	0	11	46
Esophagus (150)	0	0	0	0	0	0	0	0	0
Stomach (151)	9	5	56	0	0	0	0	4	44
Colon and rectum (153, 154)	44	27	61	8	18	1	2	8	18
Liver (155)	13	10	77	1	8	0	0	2	15
Gallbladder and extra hepatic bile duct (156)	2	2	100	0	0	0	0	0	0
Pancreas (157)	1	1	100	0	0	0	0	0	0
Lung (162)	27	21	78	2	7	2	7	2	7
Melanoma (172)	3	1	33	0	0	0	0	2	67
Skin (173)	3	1	33	0	0	0	0	2	67
Breast (174)	202	94	47	13	6	11	5	84	42
Endometrium (182)	76	22	29	40	53	9	12	5	7
Cervix (179, 180)	76	8	11	31	41	33	43	4	5
Ovary (183)	34	12	35	11	32	7	21	4	12
Bladder (188)	1	0	0	0	0	1	100	0	0
Kidney (189)	8	6	75	1	13	0	0	1	13
Brain (191)	6	5	83	0	0	0	0	1	17
Thyroid (193)	66	24	36	4	6	3	5	35	53
Lymphatic and hematopoietic tissue (200–208)	20	11	55	2	10	0	0	7	35

### Study design

The two-stage approach used in this study was designed as follows. In the first stage, for each cancer type, we compared the distribution of cancer-free survival (CFS) between the adenomyosis cohort and a matched adenomyosis-free cohort, to look for cancer types for which the cancer risk in patients with adenomyosis is different from that in the general population. The first-stage analysis was similar to previous studies [7, 15], except that we did not exclude patients who received the cancer diagnosis before the adenomyosis diagnosis. In the second stage, for each cancer type identified in the first stage, we compared the distribution of CFS between women with early-diagnosed adenomyosis and women with late-diagnosed adenomyosis, to investigate whether time of cancer onset is correlated with time of adenomyosis onset.

In the first-stage analysis, the patients who received the cancer diagnosis within 6 months after the adenomyosis diagnosis were excluded from the adenomyosis cohort, because their time of cancer diagnosis were likely to be advanced by the examination and treatment of adenomyosis, and the inclusion of these data may result in artificial difference in the distribution of CFS between adenomyosis and adenomyosis-free cohorts. Subsequently, to generate a matched adenomyosis-free cohort used in the first-stage analysis, for each patient in the adenomyosis only cohort, 10 women matched by birth year were randomly sampled from the adenomyosis-and-endometriosis-free cohort.

In the second-stage analysis, the patients who received the cancer diagnosis within 6 months before the adenomyosis diagnosis were further excluded from the adenomyosis cohort. For the patients whose adenomyosis was detected within a short time after the cancer diagnosis, the time of adenomyosis diagnosis was likely to be advanced by the examination and treatment of cancer. Subsequently, the remaining patients were divided into 34 strata by their birth year, and the medians of the age of adenomyosis onset in individual strata were calculated. In each stratum, the patients whose age-at-onset was less than the median were defined to be early-diagnosed. All early-diagnosed patients formed the early-diagnosed adenomyosis group; the remaining patients formed the late-diagnosed adenomyosis group. We would like to note that the constructed groups necessarily have identical distributions of birth year and different distributions of CFS between the early- and late-diagnosed adenomyosis groups could be observed only if the time of cancer onset is correlated with the time of adenomyosis onset.

### Statistical analysis

The Pearson chi-squared test was used to compare demographic characteristics and comorbidities between adenomyosis and adenomyosis-free cohorts. The demographic characteristics examined were geographic region, occupation, urbanization level, and monthly income; the comorbidities examined comprised chronic obstructive pulmonary disease, hypertension, hyperlipidemia, diabetes, coronary artery disease, chronic renal disease, and pelvic inflammatory disease.

In the first-stage analysis, for each cancer type, we compared the distribution of CFS in the adenomyosis cohort with that in the matched adenomyosis-free cohort by using the Cox proportional hazards model. The hazard ratio (HR) and its 95% confidence interval (CI) were estimated and adjusted for the demographic characteristics and comorbidities that were significantly different between the adenomyosis and adenomyosis-free cohorts as well as adjusted for birth year. Furthermore, the CFS curves were estimated by Kaplan-Meier approach and the difference in CFS curves between adenomyosis and adenomyosis-free cohorts was examined by the log-rank test.

In the second-stage analysis, for each cancer type identified in the first-stage analysis, we also compared the distribution of CFS in the late-diagnosed adenomyosis group with that in the early-diagnosed adenomyosis group by using the Cox proportional hazards model. The HR and its 95% CI were estimated and adjusted for birth year.

All statistical analyses were performed using SAS statistical software (version 9.4 for Windows; SAS Institute, Inc., Cary, NC, USA), and the significance level was set at 0.05. For comparison, the analytic procedures for first-stage analysis were also applied to the endometriosis cohort.

## Results

As shown in S1 Table, the LHID2000 contained data of 280,419 women born between 1951 and 1984. Of them, 249,541 (89%) had been unaffected by adenomyosis or endometriosis. Among the 30,878 (11%) women affected by adenomyosis or endometriosis, 12,447 (4.4%) women were affected by adenomyosis only, 10,962 (3.9%) women were affected by endometriosis only, and the remaining 7,469 (2.7%) women were simultaneously affected by adenomyosis and endometriosis. When stratifying the study population by birth year, the highest proportion of women affected by adenomyosis or endometriosis (15.5%) was found in the stratum of women born in 1962, the highest proportion of women affected by adenomyosis only (8.2%) was found in the stratum of women born in 1960, and the highest proportion of women affected by endometriosis only (5.2%) was found in the stratum of women born in 1977.

The results of comparisons of demographic characteristic and comorbidities between adenomyosis and adenomyosis-free cohorts and between endometriosis and endometriosis-free cohorts are summarized in Table 2 and S3 Table, respectively. As shown in these tables, all demographic characteristics and comorbidities examined were significantly different not only between the adenomyosis and adenomyosis-free cohorts but also between the endometriosis and endometriosis-free cohorts. Therefore, these variables were adjusted for in the first-stage association analysis.

**Table 2:** Comparisons of demographic characteristics and comorbidities between adenomyosis and adenomyosis-free cohorts.

Demographic characteristics and comorbidities	Adenomyosis-free		Adenomyosis		*p* value
	(n = 124,470)		(n = 12,447)		
	n	%	n	%	
Birth cohort					.99
1951–1960	46200	37.1	4620	37.1	
1961–1970	52140	41.9	5214	41.9	
1971–1980	22050	17.7	2205	17.7	
1981–1984	4080	3.3	408	3.3	
Geographic region					< 0.0001
Northern	62489	50.2	5517	44.3	
Central	24011	19.3	2414	19.4	
Southern	32899	26.4	3958	31.8	
Eastern and Islands	5069	4.1	558	4.5	
Occupation					0.0031
White collar	73005	58.6	7117	57.2	
Blue collar	37816	30.4	3956	31.8	
Retired and others	13649	11.0	1374	11.0	
Urbanization level					< 0.0001
Urban	79068	63.5	7575	60.9	
Suburban	37143	29.8	3963	31.8	
Rural	8257	6.6	909	7.3	
Monthly income, NT$					< 0.0001
≤15,840	39763	32.0	3613	29.0	
15,841–25,000	61518	49.4	6481	52.1	
≥25,001	23189	18.6	2353	18.9	
Comorbidity					
Chronic obstructive pulmonary disease	22255	17.9	2999	24.1	< 0.0001
Hypertension	18176	14.6	2474	19.9	< 0.0001
Hyperlipidemia	15532	12.5	2281	18.3	< 0.0001
Diabetes	8190	6.6	1134	9.1	< 0.0001
Coronary artery disease	6240	5.0	970	7.8	< 0.0001
Chronic renal disease	4073	3.3	570	4.6	< 0.0001
Pelvic inflammatory disease	24995	20.1	4756	38.2	< 0.0001

The distribution of cancer types in the adenomyosis cohort is summarized in Table 1. The patient numbers for cancers of the head and neck, colon and rectum, liver, lung, breast, endometrium, cervix, ovary, thyroid, and lymphatic and hematopoietic tissue were more than or equal to 10. Among these cancers, adenomyosis and cancer were detected within an interval of 6 months in high proportions of the patients with cancers of the endometrium (64.47%), cervix (84.21%), and ovary (52.94%). Similarly, distribution of cancer types in the endometriosis cohort is summarized in S2 Table. The patient numbers for cancers of the head and neck, stomach, colon and rectum, breast, endometrium, cervix, ovary, thyroid, and lymphatic and hematopoietic tissue were more than or equal to 10. Among these cancers, endometriosis and cancer were detected within an interval of 6 months in high proportions of the patients with cancers of the endometrium (52.38%), cervix (63.16%), and ovary (55.56%).

**Fig 1:**
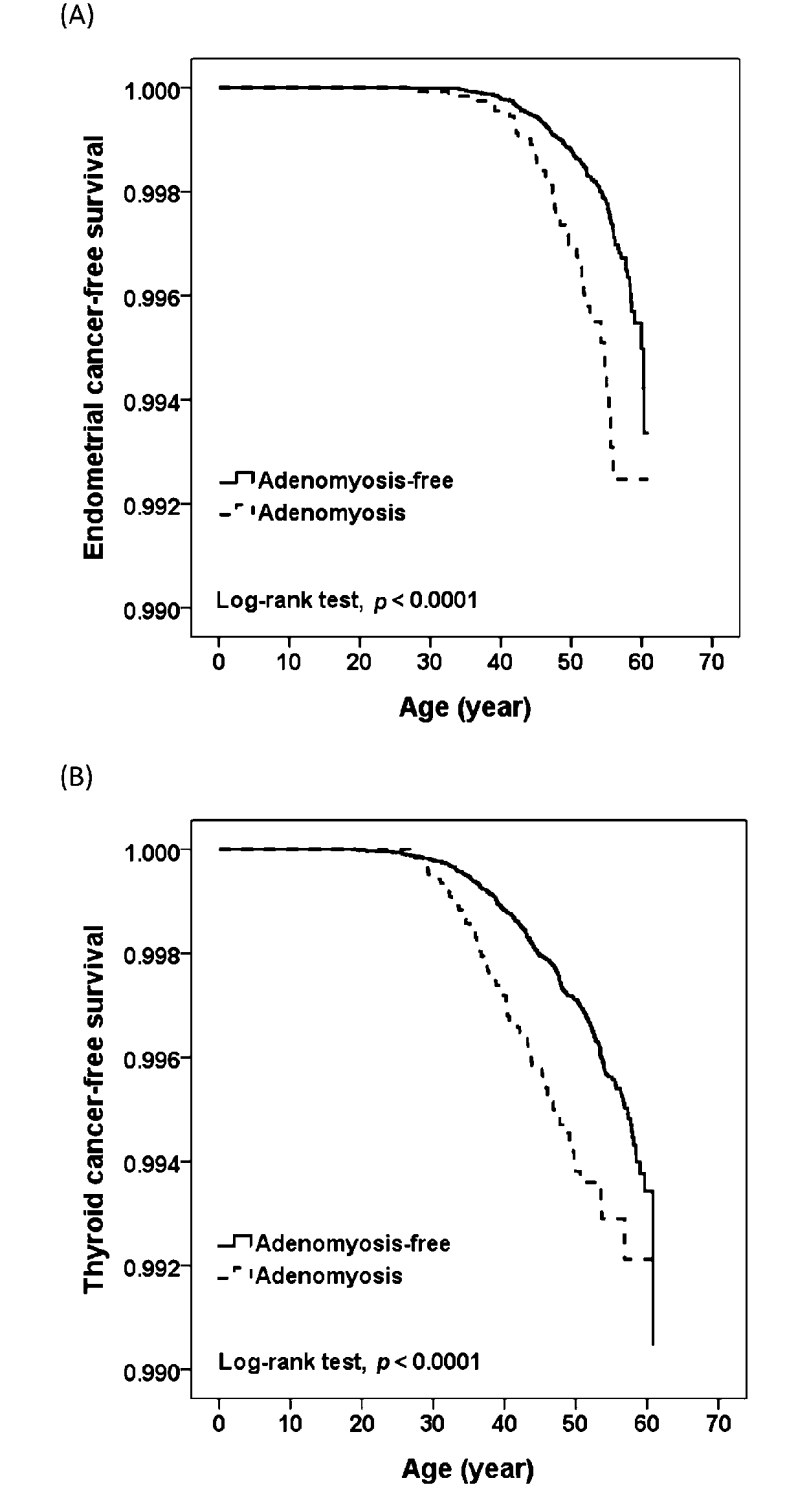
Cancer-free survival (CFS) curves in adenomyosis and adenomyosis-free cohorts. (A) CFS curves for endometrial cancer. (B) CFS curves for thyroid cancer.), for both endometrial and thyroid cancers, the CFS curve of the adenomyosis cohort significantly deviated from that of the adenomyosis-free cohort (both *p* < 0.001).

For individual cancer types, the results of comparisons of the distributions of CFS between the adenomyosis and adenomyosis-free cohorts are summarized in Table 3. As shown in Table 3, compared with the matched adenomyosis-free cohort, the adenomyosis cohort had significantly higher risks of endometrial cancer and thyroid cancer, with adjusted HRs of 2.19 (95%CI: 1.51–3.16) and 1.70 (95%CI: 1.29–2.24), respectively, as shown in Fig 1.

**Table 3:** Comparison of distribution of cancer-free survival (CFS) between adenomyosis and adenomyosis-free cohorts.

Site of cancer (ICD-9-CM)	Adenomyosis-free			Adenomyosis			HR (95% CI) ^b^
	n	Number of cancer	Rate ^a^	n	Number of cancer	Rate ^a^	
Head and neck (140–149)	124470	184	0.32	12447	24	0.41	1.27 (0.83–1.96)
Colon and rectum (153, 154)	124390	311	0.54	12439	36	0.61	1.11 (0.78–1.56)
Liver (155)	124460	104	0.18	12446	12	0.20	1.08 (0.59–1.98)
Lung (162)	124450	194	0.34	12445	25	0.43	1.20 (0.79–1.83)
Breast (174)	124340	1641	2.87	12434	189	3.22	1.12 (0.96–1.30)
Endometrium (182)	124070	151	0.26	12407	36	0.61	**2.19 (1.51–3.16)** ^c^
Cervix (179, 180)	124160	454	0.79	12416	45	0.77	0.93 (0.69–1.27)
Ovary (183)	124360	181	0.32	12436	23	0.39	1.20 (0.78–1.87)
Thyroid (193)	124430	320	0.56	12443	62	1.06	**1.70 (1.29–2.24)** ^c^
Lymphatic and hematopoietic tissue (200–208)	124450	173	0.30	12445	18	0.31	1.00 (0.61–1.63)

^a^ per 10000 person-year.

^b^ Adjusted for birth year and significant variables in Table 2.

^c^ *p* value < 0.001.

The results of comparisons of the distributions of CFS between the endometriosis and endometriosis-free cohorts are summarized in S4 Table. Compared with the matched endometriosis-free cohort, the endometriosis cohort had significantly higher risks of endometrial cancer and ovarian cancer, and the adjusted HRs were 1.89 (95%CI: 1.07–3.35) and 2.01 (95%CI: 1.27–3.16), respectively. For endometrial and ovarian cancers, the CFS curves in the endometriosis and endometriosis-free cohorts are presented in S1 Fig and significant difference in CFS curves between endometriosis and endometriosis-free cohorts was observed for both cancer types (*p* = 0.008 and 0.003, respectively).

For comparison, the ovarian CFS curves in adenomyosis and adenomyosis-free cohorts and the thyroid CFS curves in endometriosis and endometriosis-free cohorts were also estimated and shown in S2 and S3 Figs, respectively, and no significant difference was observed in both scenarios (*p* = 0.422 and 0.438, respectively).

For endometrial cancer and thyroid cancer, the results of comparisons of the distributions of CFS between women with early-diagnosed adenomyosis and women with late-diagnosed adenomyosis are summarized in Table 4. For both cancer types, no significant difference was observed. Specifically, in women with late-diagnosed adenomyosis, compared with women with early-diagnosed adenomyosis, the adjusted HRs for endometrial cancer and thyroid cancer were 0.79 (95%CI: 0.37–1.69) and 1.10 (95%CI: 0.66–1.83), respectively.

**Table 4:** Comparison of distribution of CFS between early- and late-diagnosed adenomyosis groups.

Site of cancer (ICD-9-CM)	Early-diagnosed adenomyosis			Late-diagnosed adenomyosis			HR (95% CI) ^b^
	n	Number of cancer	Rate ^a^	n	Number of cancer	Rate ^a^	
Endometrium (182)	6192	15	0.51	6192	12	0.41	0.79 (0.37–1.69) ^c^
Thyroid (193)	6213	28	0.96	6213	31	1.06	1.10 (0.66–1.83) ^c^

^a^ per 10000 person-year.

^b^ Adjusted for birth year.

^c^ *p* value > 0.05.

## Discussion

This study mainly investigated cancer risk in patients with adenomyosis by using a large nationwide health insurance database collected in Taiwan. We found that women with adenomyosis are at elevated risks of endometrial cancer and thyroid cancer. Although the association between adenomyosis and endometrial cancer has been reported by some studies [7, 11, 16], the association between adenomyosis and thyroid cancer has rarely been reported [17]. To the best of our knowledge, this is the first study providing significant evidence of this association in an ethnic Chinese population. Furthermore, we found that women with endometriosis have higher risks of endometrial cancer and ovarian cancer, and these observations are consistent with those of previous studies [5, 15, 18, 19]. The Kaplan-Meier analyses showed that significant difference in thyroid CFS curves was observed between adenomyosis and adenomyosis-free cohorts but not between endometriosis and endometriosis-free cohorts. Conversely, significant difference in ovarian CFS curves was observed between endometriosis and endometriosis-free cohorts but not between adenomoysis and adenomyosis-free cohorts. Our findings suggest that cancer types associated with adenomyosis are not identical to those associated with endometriosis.

Because women with adenomyosis are at higher risks of endometrial cancer and thyroid cancer, the corresponding HR estimated in the second-stage analysis should be less than 1 if the time of cancer onset is correlated with the time of adenomyosis onset. However, for both cancer types, no significant result was obtained. Nevertheless, the estimated HRs seemed to suggest different relationships between adenomyosis and these two cancer types.

For endometrial cancer, the estimated HR was 0.79. This finding implied that, on average, the age of endometrial cancer onset in women with early-diagnosed adenomyosis was younger than that in women with late-diagnosed adenomyosis. Some case reports of endometrial cancer arising from adenomyosis have been published [20–22], although the number of such cases is limited. Therefore, the time of endometrial cancer onset may be correlated with the time of adenomyosis onset, at least in some cases, and the nonsignificant result obtained in the current study might be attributed to the limited number of endometrial cancer cases included in the second-stage analysis.

By contrast, for thyroid cancer, the estimated HR was 1.1. This finding indicated that, on average, the age of cancer onset in women with early-diagnosed adenomyosis was not younger than that in women with late-diagnosed adenomyosis, even though women with adenomyosis are at higher risk of thyroid cancer. Although both women and men can be affected by thyroid cancer, the incidence of thyroid cancer is significantly higher in women than in men [23, 24]. So far, significant association between adenomyosis and thyroid cancer has been reported explicitly by Brinton et al. (1997) only [17]. Furthermore, Melin et al. (2007) reported a significant association between endometriosis and thyroid cancer, which was based on a sample consisting of women with endometriosis or adenomyosis [25]. Accumulating evidence indicates that some hormonal, reproductive, and genetic factors are associated with both adenomyosis and thyroid cancer. For example, a higher number of live births is associated with not only a higher risk of adenomyosis [3, 26] but also a higher risk of thyroid cancer [23, 27]. Furthermore, the expression of several genes, such as GRIM-19, CXCL12, and PAK4, is associated with not only the development of thyroid cancer [28–30] but also the pathogenesis of adenomyosis [31–33]. Therefore, the time of adenomyosis onset may be independent of the time of thyroid cancer onset, and the significant association between these two diseases observed in our first-stage analysis may be attributed to some common risk factors.

Although both adenomyosis and endometriosis have been recognized more than one century and are prevalent in women of reproductive age [34], the etiology and pathogenesis of these two diseases remain poorly understood to date [31–33, 35]. A comprehensive understanding of the relationship between adenomyosis/endometriosis and comorbidities, such as cancer, may provide new insight into the causes of adenomyosis/endometriosis [5]. For example, evidence of some genetic factors shared by endometriosis and ovarian cancer has been reported recently [36, 37]. Moreover, in a very recent study of endometriosis without cancer, several somatic cancer driver mutations were identified in ten of 39 deep infiltrating lesions and the occurrence of these mutations was unlikely by chance alone [35]. Since a number of similarities exist between adenomyosis and endometriosis [34], results of above studies may not only provide biological plausibility supporting our findings but also suggest feasible strategies for identifying causes of adenomyosis. Our findings improved the knowledge of spectrum of adenomyosis-associated cancer types and it could be beneficial to incorporate information of these associations into studies of adenomyosis as well as associated cancer types in the future.

This study had some limitations. First, as mentioned earlier in the text, the adenomyosis cohort used in this study was probably insufficiently large to obtain conclusive results in the second-stage analysis. In the future, a study based on a larger adenomyosis sample should be conducted not only to examine the correlation between time-to-onset of adenomyosis and that of associated cancer types but also to examine whether a causal relationship underlies the observed association. Second, to distinguish cancer types associated with adenomyosis from that associated with endometriosis, the patients affected by both adenomyosis and endometriosis were excluded from the current study. In addition, as shown in S1 Table, the mixed adenomyosis and endometriosis cohort (n = 7,469) was smaller than both the adenomyosis only (n = 12,447) and endometriosis only (n = 10,962) cohorts. Therefore, to investigate cancer risk in women affected by both adenomyosis and endometriosis, a larger sample should be collected in the future. Third, the NHIRD does not collect data of TNM and FIGO staging and data of histological examination for cancer patients, and any laboratory data. Therefore, the classification of type 1 and 2 endometrial cancer, the classification of histological subtypes of thyroid cancer, or the comparisons of distributions of cancer stages or profiles of hormone receptor expression between adenomyosis and adenomyosis-free cohorts was not feasible in the current study.

## Conclusions

According to the observations in our study, the spectrum of cancer types associated with adenomyosis did not seem to be identical to that of cancer types associated with endometriosis. This study revealed a significant association of adenomyosis with endometrial cancer and thyroid cancer. However, we could not provide conclusive evidence regarding whether the time-to-onset of adenomyosis is correlated with that of endometrial cancer and thyroid cancer. Further efforts are needed to reveal the exact mechanisms underlying the association observed in this study.

## Supporting information

S1 Table The proportion of women affected by adenomyosis or endometriosis in each stratum of birth year in LHID2000. (XLSX)

S2 Table Distribution of cancer types in the endometriosis cohort. (XLSX)

S3 Table Comparisons of demographic characteristics and comorbidities between endometriosis and endometriosis-free cohorts. (XLSX)

S4 Table Comparison of the distributions of cancer-free survival between endometriosis and endometriosis-free cohorts. (XLSX)

S1 Fig Cancer-free survival (CFS) curves in endometriosis and endometriosis-free cohorts. (A) CFS curves for endometrial cancer. (B) CFS curves for ovarian cancer.
(TIF)

S2 Fig Ovarian cancer-free survival curves in adenomyosis and adenomyosis-free cohorts. (TIF)

S3 Fig Thyroid cancer-free survival curves in endometriosis and endometriosis-free cohorts. (TIF)
